# Prediction of the Short-Term Risk of New-Onset Renal Dysfunction in Patients with Type 2 Diabetes: A Longitudinal Observational Study

**DOI:** 10.1155/2022/6289261

**Published:** 2022-04-20

**Authors:** Jianbo Xu, Xiaoyun Shan, Yina Xu, Yongjun Ma, Huabin Wang

**Affiliations:** ^1^Department of Clinical Laboratory, Affiliated Jinhua Hospital, Zhejiang University School of Medicine, Jinhua City, Zhejiang Province, China; ^2^Central Laboratory, Affiliated Jinhua Hospital, Zhejiang University School of Medicine, Jinhua City, Zhejiang Province, China

## Abstract

**Background:**

Studies in the past decade have reported many novel biomarkers for predicting the new-onset or progression risk of renal dysfunction in patients with type 2 diabetes (T2D) based on the genomic, metabolomic, and proteomic technologies. These novel predictive markers, however, are difficult to be widely used in clinical practice over the short term due to their high technology content, instability, and high cost. This study was aimed at evaluating the associations of clinical features and six traditional renal markers with the short-term risk of new-onset renal dysfunction in patients with T2D.

**Methods:**

This study involved 213 participants with T2D and normal renal function at baseline. The baseline levels of the albumin-to-creatinine ratio (ACR), estimated glomerular filtration rate (eGFR), alpha-1-microglobulin-to-creatinine ratio (A1MCR), neutrophil gelatinase-associated lipocalin-to-creatinine ratio, transferrin-to-creatinine ratio (UTRF/Cr), and retinol-binding protein-to-creatinine ratio (URBP/Cr) were analyzed. Multivariate logistic models were established and validated.

**Results:**

During the two-year follow-up period, 23.01% participants progressed to renal dysfunction. The basal levels of ACR, A1MCR, UTRF/Cr, and URBP/Cr were the independent risk factors of new-onset renal dysfunction (*P* < 0.05). Several logistic models incorporating clinical characteristics and these renal markers were constructed for predicting the short-term risk of new-onset renal dysfunction. Comparatively, the model including age, glycated hemoglobin (HbA1c), hypertension, ACR, A1MCR, UTRF/Cr, and URBP/Cr levels at baseline had the highest potential (C − index = 0.785, *P* < 0.001). This model was validated using the *K*-fold cross-validation method; the accuracy was 0.815 ± 0.013 in training sets and 0.784 ± 0.019 in validation sets, indicating a good consistency for predicting the new-onset renal dysfunction risk. Finally, a nomogram based on this model was constructed to provide a quantitative tool to assess the individualized risk of short-term new-onset renal dysfunction.

**Conclusion:**

The model incorporating these markers and clinical features may have a high potential to predict the short-term risk of new-onset renal dysfunction.

## 1. Introduction

The incidence of type 2 diabetes (T2D) in the Chinese general adult population is nearly 11.2%, and it continues to increase every year [1]. Diabetic kidney disease (DKD) accounts for nearly 40% of diabetes cases [2]; it has become the main serious complication of diabetes and one of the leading causes of end-stage renal disease [3]. Patients with DKD have higher risk for hospitalization, morbidity, and mortality [4, 5].

Effective prevention and early treatments can significantly improve the onset and clinical prognosis of DKD [6]; nevertheless, interventions in the later stage can only limit the damage [7].

At present, there is a lack of studies focusing on predicting the short-term risk of renal dysfunction in patients with T2D [6]. An accurate ability to predict the short-term risk of renal impairment may assist in the timely administration of interventions that can prevent or delay the progression towards DKD [6, 8]. Therefore, it is essential to stratify the patients at different risk levels, and the short-term risk prediction for patients with high risk of DKD is urgently needed. Studies in the past decade have reported many novel biomarkers for predicting the new-onset or progression risk of DKD based on the genomic, metabolomic, and proteomic technologies [4, 9, 10]. However, it is difficult to use widely these novel predictive markers in the clinical practice over the short term due to their high technology content, instability, and high cost [11]; thus, none of these novel biomarkers is applied in the clinical practice at present. Currently, in addition to ACR and eGFR, the commonly used renal markers in the clinical practice include urinary alpha-1-microglobulin, neutrophil gelatinase-associated lipocalin (NGAL), transferrin (UTRF), and retinol-binding protein (URBP). In the past, researchers mainly reported the relationships between the separate markers and the degree of DKD in the cross-section studies [12–15]; the value of the combined analysis of these markers in predicting the short-term risk of new-onset DKD is less reported.

Consequently, we hypothesized that the combined analysis of these commonly used renal markers and clinical factors would predict the risk of renal dysfunction in patients with T2D, above and beyond albuminuria and eGFR alone. We measured these urinary renal biomarkers and analyzed their association with the short-term risk of new-onset renal dysfunction in patients with T2D.

## 2. Materials and Methods

### 2.1. Study Design and Population

**Figure 1:**
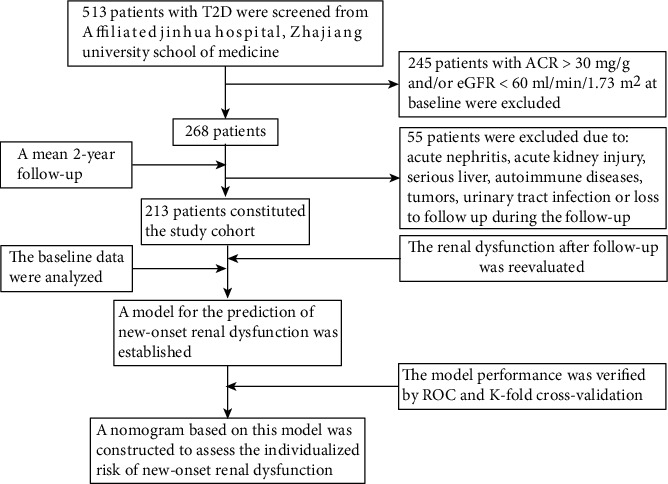
A flowchart with study design and inclusion/noninclusion criteria. T2D: type 2 diabetes; ACR: albumin-to-creatinine ratio; eGFR: estimated glomerular filtration rate; ROC: receiver operating characteristic analysis.

The longitudinal study was initiated in 2017. Among 513 patients with T2D randomly selected from the Endocrinology Department of the Affiliated Jinhua Hospital, Zhejiang University School of Medicine, between January 2017 and August 2017, there were 268 patients with ACR < 30 mg/g and eGFR > 60 mL/min/1.73 m^2^ at the baseline [13, 16]. The diagnosis of T2D was based on the “Guidelines for the Prevention and Treatment of Type 2 Diabetes in China (2013 edition)” [17]. After a mean 2-year follow-up, 55 patients were excluded from this study due to the following exclusion criteria: (1) loss to follow up; (2) patients suffered from acute nephritis or acute kidney injury during the follow-up; (3) patients suffered from serious liver, autoimmune diseases, or tumors during the follow-up period; and (4) patients suffering from urinary tract infection. Finally, 213 patients with ACR < 30 mg/g and eGFR > 60 mL/min/1.73 m^2^ at the baseline were eligible for enrollment in the present study. A flowchart with study design and inclusion/noninclusion criteria is shown in Figure 1. At the initial phase of the present study, 39.44% had hypertension and 17.84% had retinopathy among the participant cohort. Additionally, 62.91% of subjects and 37.09% of subjects were treated with oral antidiabetic drugs and oral antidiabetic drugs plus insulin, respectively; the proportion of participants with ACE inhibitor/ARB use was 20.66%. This study followed the tenets of the Declaration of Helsinki and was approved by the Ethics Committee of the Affiliated Jinhua Hospital, Zhejiang University School of Medicine.

### 2.2. Laboratory Parameters and Definitions

The first or second urine samples at baseline were collected and stored at -80°C during January 2017 to August 2017. All these specimens were thawed and recentrifuged at 400 g for 10 minutes to remove the precipitation before measurement. The levels of creatinine, albumin, alpha-1-microglobulin, NGAL, UTRF, and URBP in urine were detected using the Beckman Coulter Automatic Biochemical Analyzer (AU5800). Among them, the urinary albumin, alpha-1-microglobulin, NGAL, UTRF, and URBP levels were determined using the latex immunoturbidimetric method with Byron Diagnostics reagents (Shanghai, China). And then, these five markers were corrected according to the level of urinary creatinine; the values of ACR, alpha-1 microglobulin-to-creatinine ratio (A1MCR), NGAL-to-creatinine ratio (NGAL/Cr), UTRF-to-creatinine ratio (UTRF/Cr), and URBP-to-creatinine ratio (RBP/Cr) were calculated. The results of other laboratory measurements were collected and recorded, including serum creatinine, triglyceride, total cholesterol, high-density lipoprotein cholesterol (HDL-C), low-density lipoprotein cholesterol (LDL-C), and glycated hemoglobin (HbA1c). HbA1c was measured using the Bio-Rad D-100 analyzer and D-100 HbA1c Analytical Cartridge (High Performance Liquid Chromatography). Creatinine (Jaffe method), triglyceride (GPO-POD assay), total cholesterol (enzymatic method), HDL-C (direct assay), and LDL-C (direct assay) were all tested using the Beckman Coulter Automatic Biochemical Analyzer AU5800 and Beckman Coulter reagents. The laboratory was certified according to ISO 15189 standards, and the internal quality control procedures were used to validate the quality of data throughout the study period. All the tests were performed after internal quality control measures were passed. The clinical characteristics of the participants included in this study were collected from their electronic medical records, including age, gender, systolic blood pressure (SBP), diastolic blood pressure (DBP), height, weight, diabetes duration, hypertension (yes or no), the use of angiotensin-converting enzyme inhibitor (ACEI), angiotensin II receptor blocker (ARB), and antidiabetic drugs.

Body mass index was calculated as weight in kilograms divided by the square of height in meters. eGFR values were calculated based on the level of serum creatinine using the Xiangya equation [18]. The follow-up samples and data were collected during March 2019 and May 2020. In this study, ACR ≥ 30 mg/g was defined as albuminuria, and eGFR < 60 mL/min/1.73 m^2^ was considered as the eGFR decline. According to the prognosis of chronic kidney disease (CKD) using the GFR and albuminuria categories in the KDIGO 2020 Clinical Practice Guideline for Diabetes Management in Chronic Kidney Disease [19], the kidney function with ACR < 30 mg/g and eGFR > 60 mL/min/1.73 m^2^ was considered as low risk or no CKD; therefore, we defined that the participants with ACR < 30 mg/g and eGFR > 60 mL/min/1.73 m^2^ had normal renal function. The participants who had albuminuria and/or eGFR < 60 mL/min/1.73 m^2^ after follow-up were defined as the subjects with renal dysfunction development; the participants with normoalbuminuria and normal renal function after follow-up were considered as the subjects without renal dysfunction development.

### 2.3. Statistical Analysis

Data were summarized as mean ± standard deviation (SD), percentages, or median (interquartile, Q1–Q3), as appropriate. Differences between subjects with and without renal dysfunction development were analyzed by Student's *t*-test and Pearson's chi-squared test for normally distributed continuous variables and categorical variables, respectively. The skewed variables (such as ACR, A1MCR, NGAL/Cr, UTRF/Cr, and URBP/Cr) were subjected to log transformation to improve normality before the comparison analyses. At present, a lot of clinical prediction models are used in clinical studies, such as logistic regression, linear regression, Poisson regression, decision tree, bagging regression, random forest, and support vector machine. Logistic regression is a multivariate analysis method to evaluate the relationships between dichotomous observations and influential factors. When the dependent variable is a binary variable and the number of the independent variables is not too many in a clinical study, logistic regression is recommended. However, if the independent variables contain many classification variables, then various methods of machine learning can be tried, such as decision tree, bagging, random forest, support vector machine, and neural network. In the present study, logistic regression was used to establish the model for predicting short-term risk of renal dysfunction development. The C-indexes of the models were assessed by performing receiver operating characteristic (ROC) curve analyses, and the best model was selected out by comparing the C-indexes using the *Z* test. The accuracy validation of the model was performed using the *K*-fold cross-validation method (*K* = 5). *K*-fold cross-validation is one way to improve over the holdout method; the data is randomly and evenly split into *K* parts, and the method is repeated *k* times; each time, *k* − 1 subsets are put together to form a training set to establish the predictive model, and the other subset is used as the test set; finally, the average accuracy of the model across all *k* trials is calculated [20]. A nomogram was constructed to facilitate the use of the predictive model in clinical practice. A two-tailed *P* value < 0.05 was considered to indicate statistical significance. In this study, all the analyses and creation of graphs were performed with GraphPad Prism 8 software and R software (3.6.4 version).

## 3. Results

### 3.1. Participant Characteristics

All of the participants had normoalbuminuria and normal renal function at baseline. The average age was 57.86 ± 12.08 years; the mean follow-up time was 26.70 ± 7.59 months. Albuminuria developed in 19.72% of subjects, eGFR declined in 5.16% of subjects, and a total of 23.01% (49) of participants developed renal dysfunction during follow-up, of which 43 subjects had moderately increased risk, 4 subjects had high risk, and 2 subjects had very high risk according to the prognosis of CKD via GFR and albuminuria categories [19]. The baseline characteristics of the participants included in this study are listed in Table 1. The level of HbA1c in participants with renal dysfunction development was significantly higher than that in subjects without renal dysfunction development (8.90 ± 2.68 vs. 7.83 ± 1.87, *P* = 0.011). However, the other clinical characteristics had no statistical differences between these two groups, including age, gender, diabetes duration, the levels of SBP, DBP, serum creatinine, triglyceride, total cholesterol, HDL-C, and LDL-C. It is worth noting that although no significant difference was found in the prevalence of hypertension between these two groups (*P* = 0.059), the prevalence of hypertension in participants with renal dysfunction development was higher than that in subjects without renal dysfunction development (51.02% vs. 35.98%).

### 3.2. Comparison of Baseline Renal Markers between Subjects with and without Renal Dysfunction Development

**Figure 2:**
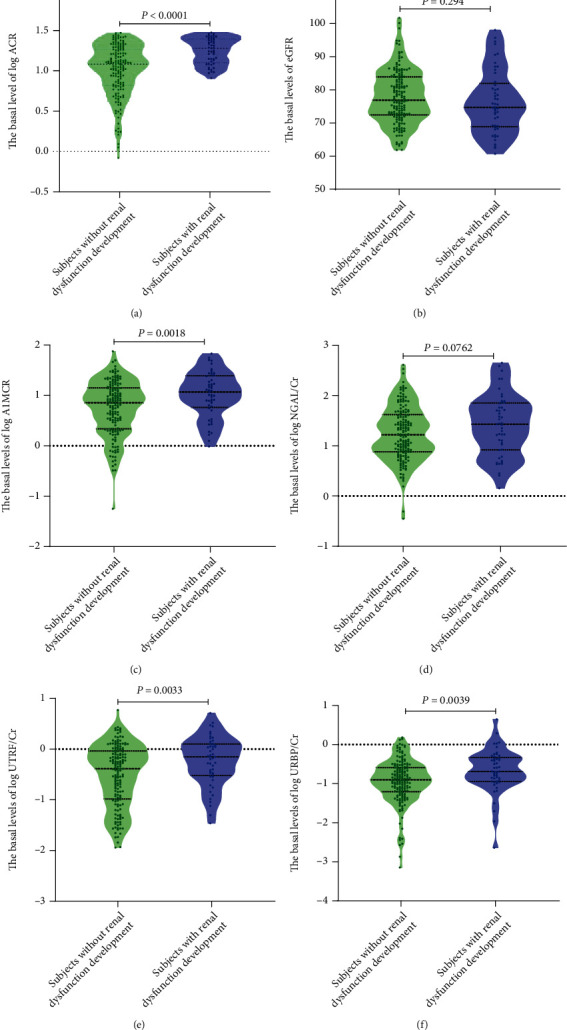
The violin plots of six markers at baseline, stratified by the development of renal dysfunction. ACR: albumin-to-creatinine ratio; A1MCR: alpha-1-microglobulin-to-creatinine ratio; eGFR: estimated glomerular filtration rate; NGAL/Cr: neutrophil gelatinase-associated lipocalin-to-creatinine ratio; UTRF/Cr: transferrin-to-creatinine ratio; URBP/Cr: retinol-binding protein-to-creatinine ratio.

The basal levels of the traditional renal markers were compared between participants with and without renal dysfunction development (Figure 2). The levels of log ACR, A1MCR, UTRF/Cr, and URBP/Cr at baseline in subjects with renal dysfunction development were obviously higher than those in participants without renal dysfunction development (*P* < 0.05). Comparatively, the most significant difference was found in log ACR levels between these two groups (*P* < 0.0001). Nevertheless, no statistical differences of both eGFR and log NGAL/Cr levels at baseline were found between these two groups, indicating the poor correlations of the basal levels of eGFR and NGAL/Cr with the short-term risk of the new-onset renal dysfunction.

### 3.3. Association of the Baseline Renal Markers with Renal Dysfunction Development Risk

These four markers which were significantly different between two groups in Figure 2 were utilized to analyze the association with the short-term risk of new-onset renal dysfunction using logistic regression analyses (Table 2). All the baseline levels of ACR, A1MCR, UTRF/Cr, and URBP/Cr were significantly associated with the risk of renal dysfunction in the univariate logistic regression analysis. Then, the clinical features for the univariate analysis (*P* < 0.1) in Table 1 were incorporated into the multivariate logistic regression analysis as the confounding factors, including age, HbA1c, and hypertension. The basal levels of ACR, A1MCR, UTRF/Cr, and URBP/Cr were still the statistically independent risk factors after adjusting for the three clinical confounding factors.

### 3.4. Establishment and Validation of the Model for Predicting the Development Risk of Renal Dysfunction

**Figure 3:**
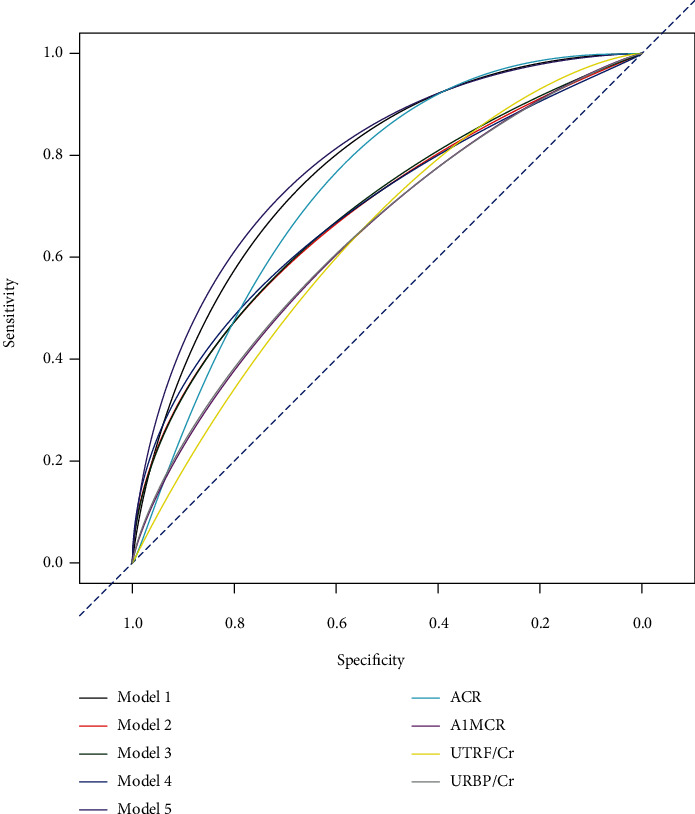
The smooth ROC curves of different models for predicting the risk of new-onset renal dysfunction. Model 1: combined analysis of age, HbA1c, hypertension, and ACR; Model 2: combined analysis of age, HbA1c, hypertension, and A1MCR; Model 3: combined analysis of age, HbA1c, hypertension, and UTRF/Cr; Model 4: combined analysis of age, HbA1c, hypertension, and URBP/Cr; Model 5: combined analysis of age, HbA1c, hypertension, ACR, A1MCR, UTRF/Cr, and URBP/Cr. ACR: albumin-to-creatinine ratio; A1MCR: alpha-1-microglobulin-to-creatinine ratio; UTRF/Cr: transferrin-to-creatinine ratio; URBP/Cr: retinol-binding protein-to-creatinine ratio.

**Figure 4:**
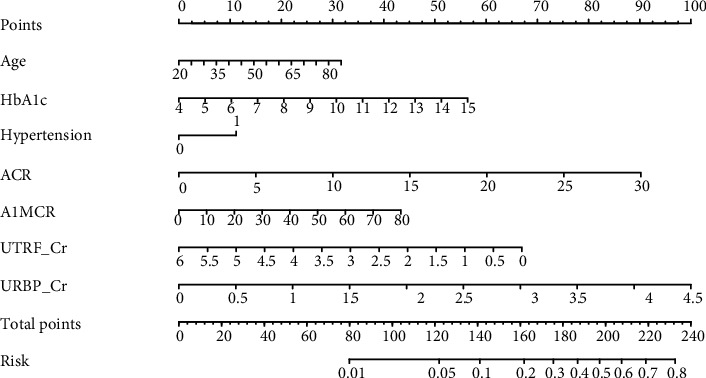
Nomogram for prediction of new-onset renal dysfunction risk. Hypertension 0: patients without hypertension; Hypertension 1: patients with hypertension; ACR: albumin-to-creatinine ratio; HbA1c: glycated hemoglobin; A1MCR: alpha-1-microglobulin-to-creatinine ratio; UTRF_Cr: transferrin-to-creatinine ratio; URBP_Cr: retinol-binding protein-to-creatinine ratio.

The baseline levels of ACR, A1MCR, UTRF/Cr, and URBP/Cr were separately incorporated with the three clinical factors (age, HbA1c, and hypertension) to establish logistic regression models for predicting the short-term risk of new-onset renal dysfunction (Model 1~Model 4, respectively). The model based on the combination analysis of these four renal markers and three clinical factors was also constructed (Model 5). The C-indexes of different models for prediction of the development risk of renal dysfunction were analyzed and compared (Figure 3 and Table 3). To a certain extent, the addition of the three clinical factors to a model with the separate renal markers improved the short-term risk prediction of renal dysfunction, although no significantly statistical differences were found between the corresponding groups (*P* > 0.05). Comparatively, Model 5 (C − index = 0.785) had a higher potential to predict the renal dysfunction risk than the other models, although the difference was not so obvious compared with Model 1. Then, the accuracy of Model 5 was validated by performing the *K*-fold cross-validation method (*K* = 5); it was 0.814 ± 0.013 in the training data and 0.784 ± 0.019 in the test data, indicating a good consistency between the predicted outcomes estimated using Model 5 and the actual outcomes. Finally, a nomogram based on Model 5 was constructed to provide a quantitative tool to assess the individualized risk of the short-term risk of new-onset renal dysfunction (Figure 4).

## 4. Discussion

In the present study with 2-year follow-up, the basal levels of ACR, A1MCR, UTRF/Cr, and URBP/Cr were each independently associated with the short-term risk of new-onset renal dysfunction. These associations remained robust even after adjustment for the clinical confounding factors, including age, HbA1c, and hypertension. Various models for predicting the risk of renal dysfunction development were constructed. Finally, we found that the model incorporating the basal levels of ACR, A1MCR, UTRF/Cr, URBP/Cr, age, HbA1c, and hypertension had the comparatively high potential to predict the short-term risk of new-onset renal dysfunction.

DKD is a complex, multifactorial syndrome that is driven by a heterogeneous set of pathophysiological processes [21, 22]. Thus, it is unlikely that a separate biomarker can capture all of the various pathophysiological processes that lead to DKD development [7]. Although the understanding of DKD pathogenesis has been improved in recent years, the risk prediction and diagnosis of DKD still largely rely on the traditional and typical biomarkers such as albuminuria and eGFR levels. However, albuminuria and eGFR are only modestly useful for risk prediction of renal insufficiency [23]. Despite researches having discovered some candidate biomarkers and improved the risk prediction and/or diagnosis for DKD [24], it is hard to popularize these novel biomarkers in clinical practice over the short term due to their high technology difficulty and high cost. Thus, the application values of the traditional renal markers in risk prediction of renal dysfunction among patients with T2D should also receive more attention.

In the present study, in addition to ACR and eGFR, four commonly used renal markers were also measured and analyzed, including urinary alpha-1-microglobulin, NGAL, UTRF, and URBP. Alpha-1-microglobulin is a 27 kDa glycoprotein which is filtered freely by the glomeruli and almost reabsorbed by the proximal tubular; it is a sensitive biomarker of renal tubular damage [25]. Previous studies reported that urinary alpha-1-microglobulin was associated with the severity and control of diabetes and was directly related to the degree of albuminuria, implying that it was a good biomarker of the severity of renal dysfunction in type 2 diabetic patients [25, 26]. The findings were in accordance with the results within our study. The baseline level of A1MCR was significantly different between subjects with and without renal impairment. NGAL was also a sensitive marker of tubular impairment. Some cross-sectional studies had found the increased levels of urinary NGAL in patients with normoalbuminuria and diabetes, indicating usefulness of NGAL as a biomarker of early DKD [27, 28]. However, compared to A1MCR, the baseline levels of NGAL/Cr did not show a statistically significant association with the short-term risk of renal dysfunction in the present study. This finding was consistent with a previous study conducted, as A1MCR was more significantly associated with renal insufficiency defined by ACR and/or eGFR compared with NGAL/Cr [13]. Furthermore, previous data had demonstrated that UTRF and URBP levels were significantly correlated with the degree of albuminuria and could be considered as the early indicators of renal damage in patients with T2D despite normoalbuminuria [14, 15]. Similar results were found in this study; the basal levels of UTRF and URBP were significantly associated with the new-onset renal dysfunction risk in patients with T2D.

In the present study, in addition to these traditional renal markers, the clinical variables for the univariate analysis (*P* < 0.1) were also incorporated to estimate the short-term risk of new-onset renal dysfunction in patients with T2D, including age, HbA1c, and hypertension. A retrospective cohort study conducted by Dr. Dorajoo et al. [6] demonstrated that the HbA1c and the presence of hypertension were the important clinical factors for new-onset albuminuria prognostication. This finding was in accordance with the result in this study. Additionally, considering the prediction efficiency and cost-effectiveness (no cost for collecting age and hypertension data), although no significantly statistical differences were found between corresponding groups, the addition of the three clinical factors to the model might be more beneficial to patients compared with the analyses of renal markers alone.

Several potential limitations of this study deserve mention. First, each kidney marker was tested only once at both baseline and during follow-up, and the definition of renal dysfunction mainly depended on the single measurement. Thus, the diurnal variation in individuals is unpredictable, and the influence of the variation on the results of this study is also unpredictable. Second, this was a single-center study with a relatively small population. Third, despite the model which was constructed by logistic regression analysis being validated using the *K*-fold cross-validation method, its performance had not been validated in external cohorts. Thus, a large-scale and well-designed study was needed to evaluate these findings of the present study.

## 5. Conclusion

In conclusion, we demonstrated that the basal levels of four commonly used renal markers, including ACR, A1MCR, UTRF/Cr, and URBP/Cr, were significantly associated with the short-term risk of new-onset renal dysfunction. This proposed model which included age, HbA1c, hypertension, ACR, A1MCR, UTRF/Cr, and URBP/Cr levels at baseline may assist in identifying patients with T2D at a short-term risk of new-onset renal dysfunction and may be valuable for communicating individualized short-term risks of renal impairment to patients with T2D who may obtain benefits from preventive interventions if and when managed early.

## Figures and Tables

**Table 1:** The baseline characteristics of the participants included in this study.

Baseline characteristics	Overall	Subjects without renal dysfunction development (*n* = 164)	Subjects with renal dysfunction development (*n* = 49)	*P* ^#^
Age (years)	57.86 ± 12.08	57.09 ± 11.26	60.45 ± 14.32	0.088
Male, *n* (%)	131 (61.50%)	105 (64.02%)	26 (53.06%)	0.166
SBP (mmHg)	132.82 ± 17.91	131.96 ± 17.53	135.67 ± 19.04	0.204
DBP (mmHg)	78.53 ± 10.46	78.46 ± 10.41	78.76 ± 10.72	0.864
Hypertension, *n* (%)	84 (39.44%)	59 (35.98%)	25 (51.02%)	0.059
Diabetes duration (years)	7.54 ± 6.12	7.55 ± 5.91	7.47 ± 6.83	0.934
ACEI/ARB use, *n* (%)	44 (20.66%)	35 (21.34%)	9 (18.37%)	0.652
Body mass index (kg/m^2^)	23.99 ± 3.33	24.02 ± 3.47	23.86 ± 2.82	0.767
HbA1c (%)	8.07 ± 2.13	7.83 ± 1.87	8.90 ± 2.68	0.011
Serum creatinine (*μ*mol/L)	75.39 ± 12.66	75.51 ± 12.01	75.02 ± 14.78	0.814
Triglyceride (mmol/L)	1.90 ± 2.10	1.87 ± 1.96	1.99 ± 2.53	0.736
Total cholesterol (mmol/L)	4.53 ± 1.12	4.52 ± 1.13	4.56 ± 1.06	0.834
HDL-C (mmol/L)	1.26 ± 0.34	1.26 ± 0.36	1.22 ± 0.27	0.403
LDL-C (mmol/L)	2.98 ± 0.82	2.96 ± 0.83	3.04 ± 0.81	0.573
eGFR (mL/min/1.73 m^2^)	77 ± 8	78 ± 8	76 ± 10	0.294
ACR (mg/g)	13.12 (8.67-20.47)	12.22 (6.79-18.53)	19.22 (13.27-24.71)	<0.001

^#^The comparison of the baseline characteristics between subjects with and without renal dysfunction development. SBP: systolic blood pressure; DBP: diastolic blood pressure; ACEI: angiotensin-converting enzyme inhibitor; ARB: angiotensin receptor blocker; HbA1c: glycated hemoglobin; HDL-C: high-density lipoprotein cholesterol; LDL-C: low-density lipoprotein cholesterol; ACR: albumin-to-creatinine ratio; eGFR: estimated glomerular filtration rate.

**Table 2:** The association of renal dysfunction development during the follow-up with the basal levels of the traditional renal biomarkers.

Variables	Univariate analyses		Multivariate analyses^a^	
	OR (95% CI)	*P*	OR (95% CI)	*P*
Basal ACR level	1.12 (1.07-1.17)	<0.001	1.12 (1.06-1.17)	<0.001
Basal A1MCR level	1.04 (1.01-1.07)	0.003	1.03 (1.01-1.06)	0.016
Basal UTRF/Cr level	1.56 (1.08-2.25)	0.019	1.48 (1.02-2.14)	0.040
Basal URBP/Cr level	3.36 (1.31-8.63)	0.012	2.64 (1.15-6.03)	0.022

^a^Adjusted for age, HbA1c level, and hypertension (yes or no) at the baseline. ACR: albumin-to-creatinine ratio; A1MCR: alpha-1-microglobulin-to-creatinine ratio; UTRF/Cr: transferrin-to-creatinine ratio; URBP/Cr: retinol-binding protein-to-creatinine ratio.

**Table 3:** The C-indexes of different models for prediction of the development risk of renal dysfunction during the follow-up.

	C-index	95% CI	*P* value
Basal ACR level	0.733	0.661~0.805	<0.001
Basal A1MCR level	0.639	0.551~0.727	0.002
Basal UTRF/Cr level	0.628	0.543~0.714	0.003
Basal URBP/Cr level	0.652	0.564~0.739	0.001
Model 1	0.768	0.697~0.839	<0.001
Model 2	0.690	0.602~0.777	<0.001
Model 3	0.696	0.609~0.784	<0.001
Model 4	0.698	0.6090~0.787	<0.001
Model 5	0.785	0.714~0.855	<0.001

Model 1: combined analysis of age, HbA1c, hypertension, and ACR; Model 2: combined analysis of age, HbA1c, hypertension, and A1MCR; Model 3: combined analysis of age, HbA1c, hypertension, and UTRF/Cr; Model 4: combined analysis of age, HbA1c, hypertension, and URBP/Cr; Model 5: combined analysis of age, HbA1c, hypertension, ACR, A1MCR, UTRF/Cr, and URBP/Cr. ACR: albumin-to-creatinine ratio; A1MCR: alpha-1-microglobulin-to-creatinine ratio; UTRF/Cr: transferrin-to-creatinine ratio; URBP/Cr: retinol-binding protein-to-creatinine ratio.

## Data Availability

Data from this study are available by request to the corresponding authors.
